# High Variability in Cellular Proliferation, Gene Expression, and Cytokine Production in the Nonneoplastic Colonic Epithelium of Young *Apc^+/Min-FCCC^* Mice

**DOI:** 10.3389/fonc.2021.705562

**Published:** 2021-08-27

**Authors:** Alyssa A. Leystra, Kristen N. Harvey, Esther Kaunga, Harvey Hensley, Lisa A. Vanderveer, Karthik Devarajan, Margie L. Clapper

**Affiliations:** ^1^Cancer Prevention and Control Program, Fox Chase Cancer Center, Philadelphia, PA, United States; ^2^Biological Imaging Facility, Fox Chase Cancer Center, Philadelphia, PA, United States; ^3^Biostatistics and Bioinformatics Facility, Fox Chase Cancer Center, Philadelphia, PA, United States

**Keywords:** colorectal cancer prevention, cancer prevention, cytokine signaling, tumor initiation, chemoprevention, immunoprevention

## Abstract

An urgent need exists to identify efficacious therapeutic preventive interventions for individuals who are at high risk of developing colorectal cancer. To maximize the benefits of preventive intervention, it is vital to identify the time interval during which the initiation of a preventive intervention will lead to an optimal outcome. The goal of the present study was to determine if oncogenic events can be detected in the nonneoplastic colonic mucosa of *Apc^+/Min-FCCC^* mice prior to formation of the first adenoma, thus defining an earlier point of intervention along the cancer continuum. Tissues taken at three potential points of intervention were characterized: prior to *Apc* mutation (wild type *Apc^+/+-FCCC^* mice); after initiation but prior to colon adenoma formation (tumor-free *Apc^+/Min-FCCC^* mice); and after formation of the first colon adenoma (tumor-bearing *Apc^+/Min-FCCC^* mice). Experimentation focused on molecular processes that are dysregulated in early colon lesions: 1) cellular proliferation (proliferative index and size of the proliferative zone); 2) cellular stemness (expression of *Ascl2*, *Grem1*, *Lgr5* and *Muc2*); 3) EGFR signaling (expression of *Ereg*); and 4) inflammation (expression of *Mmp9*, *Ptsg2*, and *Reg4*, as well as secretion of 18 cytokines involved in immune activation and response). Interestingly, the nonneoplastic colonic mucosa of wild type, tumor-free *Apc^+/Min-FCCC^*, and tumor-bearing *Apc^+/Min-FCCC^* mice did not display significant differences in average epithelial cell proliferation (fold change 0.8–1.3, p≥0.11), mucosal gene expression (fold change 0.8–1.4, p≥0.22), or secretion of specific cytokines from colonic mucosa (fold change 0.2–1.5, p≥0.06). However, the level of cytokine secretion was highly variable, with many (22% of wild type, 31% of tumor-free *Apc^+/Min-FCCC^*, and 31% of tumor-bearing *Apc^+/Min-FCCC^*) mice categorized as outliers (> 1.5 x interquartile ranges below the first quartile or above the third quartile) due to elevated expression of at least one cytokine. In summary, no differences were observed in proliferation, stemness, and EGFR signaling in the colonic mucosa of wild type vs *Apc^+/Min-FCCC^* mice, with low baseline cytokine expression, prior to the formation of the first colon adenoma. The results of this study provide valuable baseline data to inform the design of future cancer prevention studies.

## Introduction

Colorectal cancer (CRC) is the third-leading cause of cancer-related mortality in both men and women in the United States, with an estimated 147,950 new cases and 53,200 associated deaths expected this year (1). An alarming rise in CRC among younger adults (age ≤ 50) is emerging; incidence and mortality have increased 22% and 13%, respectively, over the past two decades (2). These statistics underscore the need to optimize preventive strategies to reduce CRC incidence.

The efficacy of several preventive agents varies depending on the time of intervention. Preclinical studies from this group and others indicate that some preventive agents work best when given prior to the formation of gross colon tumors (3–5). For example, thymoquinone exhibited more pronounced anti-tumor efficacy in rats when administered during initiation as opposed to afterwards (3). Administration of atorvastatin to *Apc^+/Min-FCCC^* mice that were tumor-free at the time of treatment initiation completely eliminated colon microadenomas and significantly decreased colon tumor incidence. In contrast, the regimen failed to alter tumor number or incidence in mice with pre-existing tumors (4). Likewise, a vaccine against *Ascl2* decreased the number of colon microadenomas in mice when administered prior to tumor formation, but had a minimal effect when given to mice with existing tumors (5, 6). Thus, it is critical to identify the optimal time for treatment during tumorigenesis to maximize preventive efficacy.

The precise time when targetable molecular alterations arise during early colorectal carcinogenesis is not well defined. Most sporadic colon tumors are thought to arise *via* a stepwise process in which: (1) mutation of the *APC* colon tumor suppressor gene creates an ‘initiated’ epithelium that is predisposed to tumor formation; (2) loss of *APC* heterozygosity promotes the formation of an adenoma; and (3) additional mutations drive tumor progression (7, 8). In addition to mutational events, a number of other molecular changes occur at ill-defined time points before, during, or after adenoma formation. Compared to the normal epithelium, cells from early colon lesions demonstrate increased cellular proliferation (9), stemness (10–12), and EGFR (13, 14) and inflammatory signaling (15, 16). Importantly, each of these processes can be targeted with existing chemoprevention strategies. For example, treatment of *Apc^+/Min-FCCC^* mice with atorvastatin decreases expression of stem-related genes and subsequent tumor formation (4). The EGFR inhibitor erlotinib exhibits tumor-preventive efficacy in individuals with familial adenomatous polyposis, a syndrome caused by inheritance of a mutant allele of *APC* (17). Lastly, anti-inflammatory agents, including aspirin, sulindac, and celecoxib, reduce the risk of sporadic colon tumorigenesis (18). A greater understanding of the specific time when these processes are first disrupted during carcinogenesis will help define the optimal window for preventive intervention to achieve maximal efficacy.

The *Apc^+/Min-FCCC^* mouse model provides a unique opportunity to study early events in colon tumorigenesis. As in humans, adenomas arise sporadically within the colons of *Apc^Min^* mice following loss of *Apc* (19, 20), and exhibit increased cellular proliferation (21, 22), stemness (23), and EGFR (13, 24) and inflammatory signaling (25, 26). Thus, with respect to these events, the nonneoplastic colon tissue of the *Apc^+/Min-FCCC^* mouse mimics the initiated epithelium of an individual at high risk for CRC.

**Figure 1:**
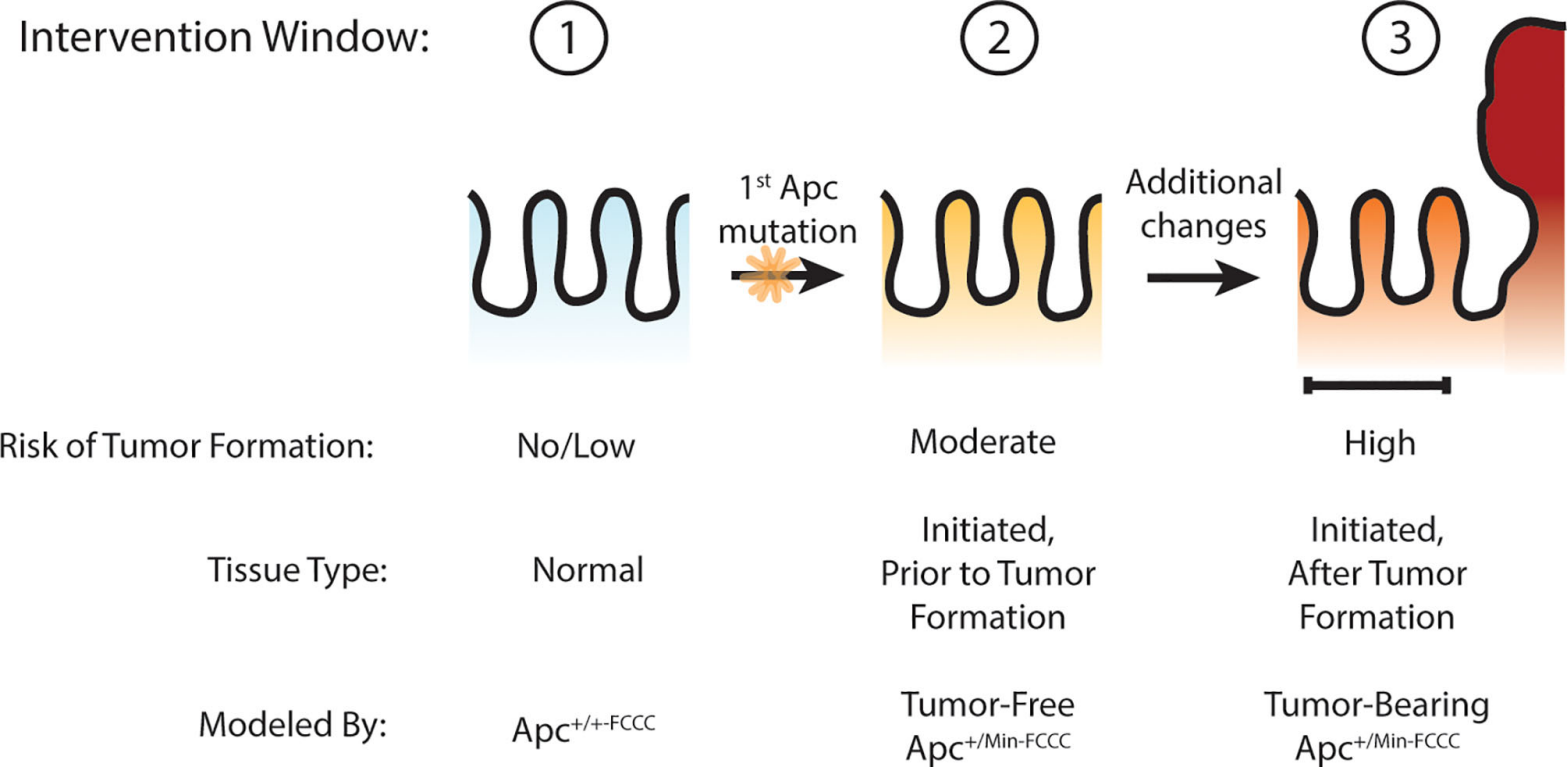
Windows of opportunity for preventive intervention for colon cancer. Three potential windows of opportunity were defined: (1) prior to tissue initiation (Apc mutation); (2) after initiation but prior to adenoma formation; (3) and after formation of the first adenoma. To examine these windows of opportunity, nonneoplastic tissue from mice representing each stage of early tumorigenesis was evaluated and pro-tumorigenic processes were characterized.

Others have hypothesized that *Apc* haploinsufficiency created by mutation of one allele of *Apc* might be sufficient to increase Wnt signaling in the colon crypt, expand the stem cell compartment, drive increased proliferation, and ultimately lead to adenoma formation (27–30). The goal of the present study was to test this hypothesis by determining if oncogenic events can be detected in the nonneoplastic colorectal epithelium that has been initiated through heterozygous mutation of *Apc*, thus defining an earlier point of intervention along the cancer continuum (**Figure 1**). Experimentation focused on molecular processes that can be targeted with existing preventive agents, including cellular proliferation (4), stemness (4), and EGFR (17) and inflammatory signaling (18). Three time points were modeled: prior to *Apc* mutation (wild type *Apc^+/+-FCCC^* mice); after initiation but prior to colon adenoma formation (tumor-free *Apc^+/Min-FCCC^* mice); and after formation of the first colon adenoma (tumor-bearing *Apc^+/Min-FCCC^* mice). A greater understanding of the timing of molecular changes during early colon tumorigenesis will aid in defining the optimal time interval (prior to initiation, after initiation but prior to formation of the first colon tumor, or after colon tumor formation) to initiate preventive interventions in high-risk individuals. In addition, the results of this study will provide valuable baseline data to inform the design of new therapeutic strategies for preventive intervention.

## Materials and Methods

### Animals

Male C57BL/6-*Apc^+/Min-FCCC^* mice (5 weeks old) were obtained from a closed colony that has been maintained at the Fox Chase Cancer Center for at least 50 generations (31). *Apc^+/Min-FCCC^* mice and *Apc^+/+-FCCC^* (wild type) littermates were provided autoclaved Rodent Breeder Diet (LabDiet #5013) and water ad libitum. At weaning, mice were assigned to cages by block randomization of litters, and switched to autoclaved 2018 Teklad 18% Protein/Extruded Global Rodent Diet (Envigo #2018SX) and autoclaved, double-distilled water for the duration of the study. Animals were housed in ventilated cages and maintained at 70 ± 2°F and 40-70% relative humidity with a 12-hour light/dark cycle. All animal experiments were reviewed and approved by the Institutional Animal Care and Use Committee at the Fox Chase Cancer Center.

### Colonoscopy

5-week-old mice weighing >15 g (33/36 *Apc^+/Min-FCCC^* mice and 9/10 wild type mice) were evaluated for colon tumors *via* colonoscopy, as described previously (4, 32). Colon tumors were detected in 3% (1/33) of the *Apc^+/Min-FCCC^* mice. As expected, no colon tumors were found in wild type mice.

### Necropsy

At ~8 weeks of age, mice were injected with 100 mg/kg BrdU and euthanized one hour later by CO_2_ inhalation. Colons were excised, opened lengthwise, washed with PBS and examined grossly. The number, location, and size (length and width as measured with calipers) of each colon tumor was recorded. Colon tumor volume was calculated as a simplified approximation of an ellipsoid as follows:

$$volume=\frac{\text{length }\times \;{\text{width}}^{2}}{2}$$

Total tumor burden per mouse was calculated as the sum of all tumor volumes within the colon of that animal.

The distal portion of the colon (2.2 cm) was cut in half lengthwise and each half was divided into 2 mm sections (**Supplemental Figure 1**). Alternating pieces from one side were snap frozen in liquid nitrogen or placed in media for *ex vivo* cytokine analysis. Alternating pieces from the other side were embedded in OCT and frozen on dry ice or fixed in 4% paraformaldehyde for two days, equilibrated in a series of sucrose in 1x PBS (30% sucrose followed by 50% sucrose), embedded in OCT and frozen on dry ice. Any tissue section that contained a macroscopic colon tumor was excluded from molecular analysis.

### BrdU Staining

OCT blocks of PFA-fixed tissue were cut (10 µM sections), thawed, and permeabilized in ice-cold methanol for 10 minutes. Antigen retrieval was performed with Citrate buffer (pH 6.0, 0.005% Tween) in a steamer for 20 minutes and cooled at room temperature for 40 minutes. Blocking, primary staining (rat anti-BrdU, 1:500; clone ICR1 Abcam ab6326), and secondary staining were completed with the VECTASTAIN ABC AP Kit, Rat IgG (Vector Laboratories), according to the manufacturer’s instructions. Sections were stained with DAB for 1-5 minutes, counterstained with hematoxylin, dehydrated in an ethanol series (70-100% ethanol), and cover slipped.

The total number of nuclei (stained and unstained) were counted from the right half of 6-20 full length crypts per mouse. The proliferative index was calculated as the proportion of stained nuclei normalized to the number of total nuclei in each crypt column (33). The size of the proliferative zone was calculated as the height of the highest BrdU+ cell normalized to the total height of the crypt column (33).

### RT-qPCR

OCT blocks of frozen tissue were cut (10 µM sections) and thawed. The mucosal surface was microdissected from tissue sections using a razor blade. Total RNA was extracted from the microdissected tissue using the PicoPure™ RNA Isolation Kit (ThermoFisher, #KIT0214) according to the manufacturer’s instructions. Reverse transcription was performed using the High-Capacity cDNA Reverse Transcription Kit (Applied Biosystems, Ref 4374966) per the manufacturer’s instructions. Quantitative PCR from the resulting cDNA was performed with iTaq Universal SYBR Green Supermix (Biorad). Amplification products were monitored using an ABI7900 Sequence Detection System and quantified using the comparative ^ΔΔ^Ct method. Primer sequences are available in **Supplemental Table 1**.

### *Ex Vivo* Cytokine Analysis

Tissues for *ex vivo* cytokine analyses were weighed and placed in 200 µL of RPMI containing 10% FBS, 100 U/mL Penicillin, and 100 µg/mL Streptomycin at 37°C. After 24 h, media was collected and spun at >16,000 x g at 4°C for 15 minutes. The supernatant was stored at -80°C until the time of analysis.

A Mouse Custom ProcartaPlex 18-plex chip (LifeTech, Assay ID MXGZFVD) was used to simultaneously assess the concentration of GM-CSF, GROα, IFNγ, IL-1β, IL-2, IL- 4, IL-5, IL-6, IL-9, IL-10, IL-12p70, IL-13, IL-17α, IL-18, IL-22, IL-23, IL-27, and TNFα in the supernatant from *ex vivo* cultures. Samples were loaded onto the chip according to the manufacturer’s instructions and analyzed using the Bioplex100/200 (Biorad).

### Statistical Analyses

Mann-Whitney, Welch’s t-test, and linear regression tests were used as indicated throughout the text. All tests were two-sided and employed a type I error of 5% to determine significance. Outliers were defined as data points that were > 2 standard deviations from the mean in gene expression data or > 1.5 x interquartile ranges below the first quartile or above the third quartile in cytokine expression data. P-values from the comprehensive cytokine panel were adjusted to account for multiple testing using the Benjamini-Hochberg false discovery rate approach; both uncorrected and corrected values are reported. The R statistical software, including the dplyr package, was used to complete all analyses (34, 35).

## Results

### Timing of Macroscopic Tumor Formation in *Apc^+/Min-FCCC^* Mice

**Figure 2:**
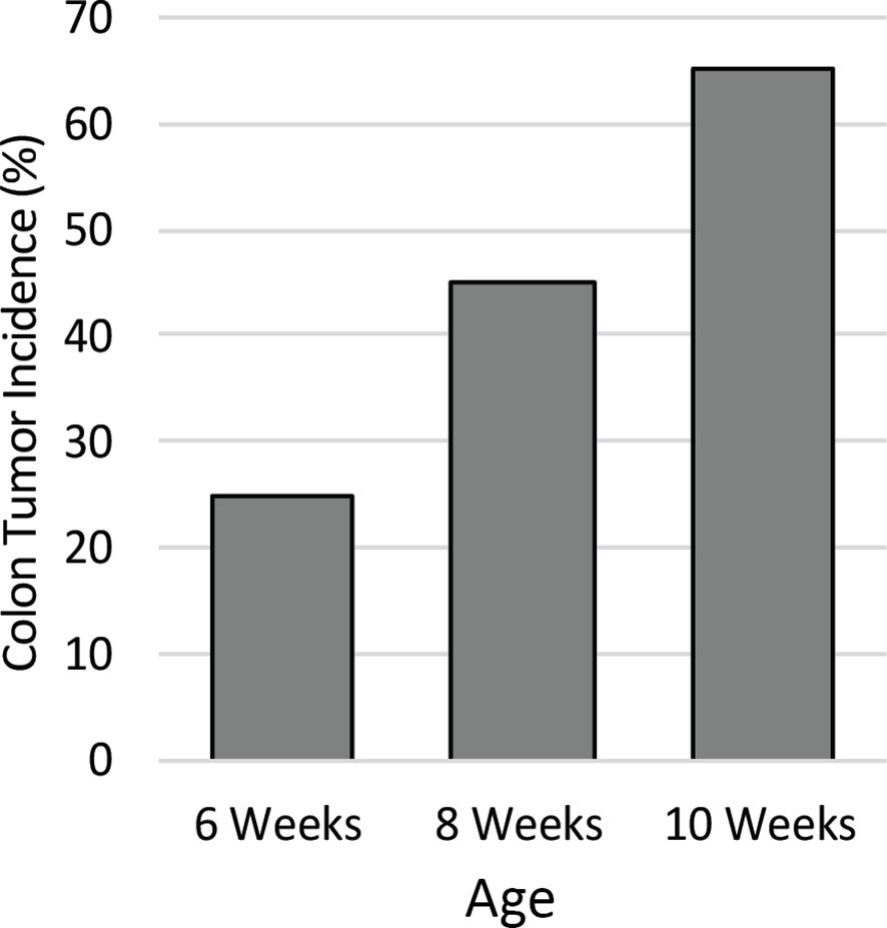
Colon tumor incidence in *Apc^+/Min-FCCC^* mice over time. Animals (n=20) assessed by colonoscopy exhibited a linear increase in colon tumor incidence from 6 to 10 weeks of age.

To identify the ideal age of mice to use in this study, a pilot study was conducted to characterize colon tumor incidence over a short time course. The colons of twenty male *Apc^+/Min-FCCC^* mice were examined by colonoscopy at 6, 8, and 10 weeks of age. Colon tumor incidence increased linearly over time; 25% of the mice had at least one colon tumor at 6 weeks of age, 45% by 8 weeks of age, and 65% at 10 weeks of age (**Figure 2**). Based on the results of the time course, 8-week-old mice were selected for further evaluation, as sexual maturity had been achieved and macroscopic colon tumors were identified in <50% of these animals.

### Animals

*Apc^+/Min-FCCC^* (n=36) and wild type (n=10) male mice were enrolled in the study. To standardize the impact of cage environment, animals were assigned to cages by block randomization of litters by 5 weeks of age. *Apc^+/Min-FCCC^* and wild type mice were cohoused.

Mouse weight represents one surrogate of overall health of the animal. To examine whether a single mutation in the *Apc* gene caused a difference in body weight, animals were weighed at 8 weeks of age. On average, *Apc^+/Min-FCCC^* mice weighed slightly less than wild type mice; *Apc^+/Min-FCCC^* weighed 21.5 ± 0.3 g (Mean ± S.E.M.) whereas wild type mice weighed 22.9 ± 0.2 g (Mean ± S.E.M.; Mann-Whitney p=0.04; **Supplemental Figure 2**).

At the time of euthanasia, colons were examined grossly to identify potential tumors. As seen in 8-week-old animals in the time course study, approximately half (42%; 15/36) of *Apc^+/Min-FCCC^* mice and 0% (0/10) of wild type mice had macroscopic colon adenomas. Mice with tumors had an average of 1.6 ± 1.0 (Mean ± S.D.; Range 1-4) colon tumors per animal. As expected, tumors arose primarily in the distal and medial colon (**Supplemental Figure 3**).

Notably, factors associated with colon tumorigenesis vary naturally along the length of the colon, including cellular proliferation (36), as well as the expression of stem cell-associated genes (28) and inflammatory cytokines (37). To control for this natural variation, all comparisons of these factors among different mouse groups were made using tissue from the distal colon, as this region is: 1) where >20% of colon tumors eventually form in *Apc^+/Min-FCCC^* mice (**Supplemental Figure 3**) can be evaluated by colonoscopy during preclinical chemo- and immunoprevention studies (4, 38, 39).

### Analysis of Cellular Proliferation in the Nonneoplastic Colonic Epithelium of *Apc^+/Min-FCCC^* and Wild Type Mice

**Figure 3:**
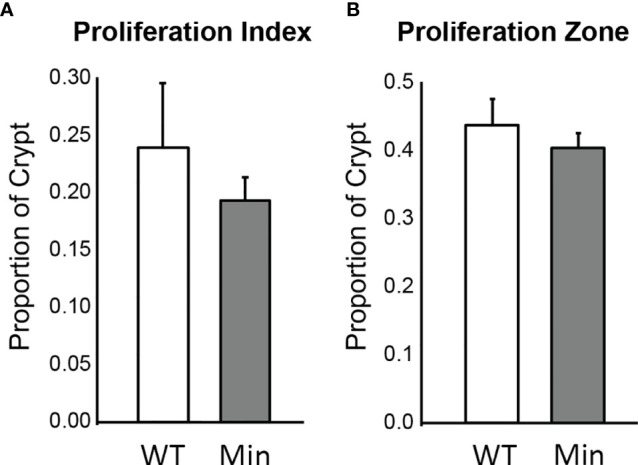
Epithelial cell proliferation is similar in the distal colon of *Apc^+/Min-FCCC^* (‘Min’; n=12) *vs. Apc^+/+-FCCC^* wild type (‘WT’; n=4) mice. Bars represent mean ± S.E.M. **(A)** Fold change in mean proliferative index = 0.8; Welch’s t-test p = 0.55. **(B)** Fold change in mean size of the proliferative zone = 0.9; Welch’s t-test p = 0.56.

The rate of cellular proliferation in the initiated epithelium of *Apc^+/Min-FCCC^* mice was compared to that of the uninitiated epithelium of wild type mice. Proliferating cells were labeled *via* BrdU incorporation and a 2 cm segment of the distal colon was examined (**Supplemental Figure 1**). Both the proliferative index (the proportion of epithelial cells within each crypt that are actively proliferating) and the size of the proliferative zone (the region at the base of the crypt that contains proliferating cells) within nonneoplastic colonic crypts were quantified. No significant difference in the proliferative index of nonneoplastic colonic crypts of *Apc^+/Min-FCCC^* and wild type mice was detected (Mean ± S.E.M. - 19 ± 6% and 24 ± 6%, respectively; Welch’s t-test p = 0.55) (**Figure 3**). Likewise, the size of the proliferative zones within the nonneoplastic crypts of *Apc^+/Min-FCCC^* and wild type mice was comparable (Welch’s t-test p = 0.56, **Figure 3**); the average size of the proliferative zone of nonneoplastic crypts from *Apc^+/Min-FCCC^* mice was 40 ± 2% (Mean ± S.E.M.), compared to 44 ± 4% for wild type mice. Thus, cellular proliferation is similar in the nonneoplastic colonic epithelium of *Apc^+/Min-FCCC^* and wild type animals.

### Expression of Adenoma-Associated Genes in the Nonneoplastic Colonic Mucosa of *Apc^+/Min-FCCC^* and Wild Type Mice

To determine whether gene transcription was impacted by a single *Apc* mutation in the nonneoplastic mucosa of young *Apc^+/Min-FCCC^* mice, a panel of genes known to be modulated during early tumorigenesis was selected for analysis. The gene set included stem-related genes [*Ascl2* (12, 40, 41), *Grem1* (42, 43), and *Lgr5* (40, 44)], a differentiation-associated gene [*Muc2* (44, 45)], inflammation-associated molecules [*Mmp9* (46), *Ptsg2* (47), and *Reg4* (48, 49)]; and an EGFR ligand [*Ereg* (13, 50)]. Relative RNA expression levels in tissues from *Apc^+/Min-FCCC^* and wild type mice were compared by qPCR. Overall, the mean relative expression of all genes analyzed was comparable in the nonneoplastic mucosa of *Apc^+/Min-FCCC^* and wild type mice (fold change 0.9 – 1.4, Welch’s t-test p≥0.22; **Table 1**; **Supplemental Table 2**).

**Table 1:** Fold change in mean gene transcription in the initiated colonic mucosa of *Apc^+/Min-FCCC^* (Min) *vs* wild type (WT) mice after removing outliers.

Gene	Pathway	Fold Change (Min/WT)	P-value*
*Ascl2*	Cellular Stemness	1.18	0.52
*Ereg*	EGFR signaling	0.93	0.82
*Grem1*	Cellular stemness	0.91	0.46
*Lgr5*	Cellular stemness	1.38	0.22
*Mmp9*	Inflammation	1.10	0.66
*Muc2*	Cellular differentiation	1.10	0.33
*Ptgs2*	Inflammation	0.97	0.89
*Reg4*	Inflammation	0.90	0.62

*Two-sided Welch’s t-test.

### Cytokine Expression in the Nonneoplastic Colon Tissue of Mice

**Figure 4:**
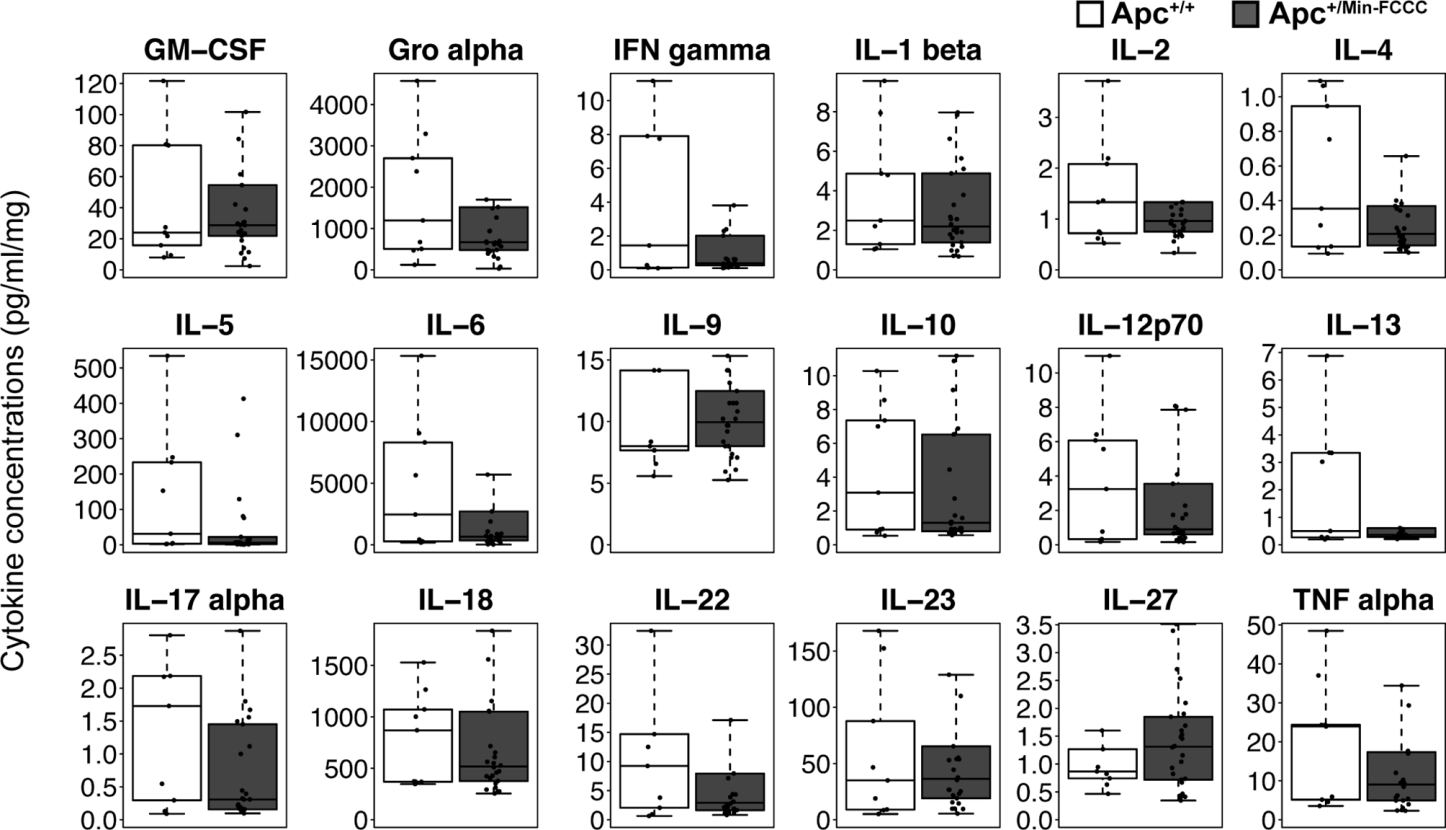
Cytokines involved in Th1, Th2, Th9, and Th17 signaling in colon tissues from *Apc^+/Min-FCCC^* and *Apc^+/+-FCCC^* wild type mice. Boxplots depict cytokine concentration in media (pg/ml) normalized to mass of colon tissue (mg) from *Apc^+/+-FCCC^* wild type mice (white; n=9) and *Apc^+/Min-FCCC^* mice (gray; n=26). Outlying data points (>3rd quartile + 1.5 x interquartile range) are omitted; all data points are shown in **Supplemental Figure 4**. Median fold change = 0.2 – 1.5; Mann Whitney p≥0.17; p=1.00 after adjusting for multiple comparisons.

Inflammation is associated with an increased risk of colon tumorigenesis. As observed in humans, inflammatory cytokine production can increase colon tumor risk in *Apc^+/Min^* mice (15, 51, 52). Thus, the basal levels of cytokines involved in Th1, Th2, Th9, and Th17 immune activation and response were evaluated in nonnoneoplastic colon tissue from *Apc^+/Min-FCCC^* mice and compared to those of the uninitiated mucosa of wild type mice at 8 weeks of age. Most (14/18) cytokines were secreted at lower median levels in colon tissue from *Apc^+/Min-FCCC^ vs*. wild type mice, although no comparisons reached statistical significance (fold change 0.2 – 1.5, Mann-Whitney p≥0.32; **Table 2** and **Figure 4**).

**Table 2:** Median fold change in secretion of cytokines involved in Th1, Th2, Th9, and Th17 signaling from nonneoplastic colon tissue from *Apc^+/Min-FCCC^ vs*. normal colon tissue from wild type mice.

Cytokine	Fold change	P-value*
GM-CSF	1.20	0.59
Gro alpha	0.56	0.49
IFN gamma	0.28	0.72
IL-1 beta	0.88	0.70
IL-2	0.72	0.92
IL-4	0.59	0.50
IL-5	0.21	0.25
IL-6	0.26	0.64
IL-9	1.24	0.57
IL-10	0.42	0.66
IL-12p70	0.28	0.75
IL-13	0.72	0.88
IL-17a	0.18	0.17
IL-18	0.60	0.75
IL-22	0.32	0.56
IL-23	1.04	0.51
IL-27	1.51	0.56
TNF alpha	0.38	0.52

*Uncorrected Mann-Whitney test; p=1.00 after correcting for multiple comparisons.

A high degree of variability in cytokine expression was observed among the mice. In general, the interquartile range and median absolute deviation of cytokine expression was greater in wild type mice, while the number of outliers (> 1.5 x interquartile ranges below the first quartile or above the third quartile) was greater in *Apc^+/Min-FCCC^* mice (**Table 3** and **Supplemental Figure 4**). Variability was high even among animals from the same litter, batch, or cage (**Supplemental Figure 5**). Although production of inflammatory cytokines is increased in obese animals (53–55), cytokine expression did not correlate with the weight of the animals in this study (linear model R^2^ ≤ 0.08; p≥0.13; **Supplemental Figure 6**). Thus, no single genetic or environmental factor was identified that accounted for the inter-mouse variability in cytokine expression.

**Table 3:** Intra-group variability in secretion of cytokines involved in Th1, Th2, Th9, and Th17 signaling from initiated colon tissues from *Apc^+/Min-FCCC^* mice vs normal colon tissues from wild type mice.

Cytokine	Median Concentration (pg/ml/mg)		MAD* Expression		Proportion of mice with outlying expression^†^	
	Wild type	*Apc^+/Min-FCCC^*	Wild type	*Apc^+/Min-FCCC^*	Wild type	*Apc^+/Min-FCCC^*
GM-CSF	24	29	22	17	0% (0/9)	15% (4/26)
Gro alpha	1195	670	1591	461	0% (0/9)	19% (5/26)
IFN gamma	1.44	0.40	1.99	0.33	11% (1/9)	15% (4/26)
IL-1 beta	2.5	2.2	2.2	1.6	0% (0/9)	4% (1/26)
IL-2	1.33	0.96	1.06	0.40	0% (0/9)	19% (5/26)
IL-4	0.35	0.21	0.39	0.13	0% (0/9)	12% (3/26)
IL-5	31.2	6.6	43.2	7.6	0% (0/9)	23% (6/26)
IL-6	2454	649	3369	609	0% (0/9)	19% (5/26)
IL-9	8.0	9.9	2.1	3.3	11% (1/9)	8% (2/26)
IL-10	3.1	1.3	3.8	0.8	0% (0/9)	4% (1/26)
IL-12p70	3.25	0.90	4.32	0.95	0% (0/9)	19% (5/26)
IL-13	0.50	0.36	0.45	0.15	0% (0/9)	23% (6/26)
IL-17a	1.73	0.31	1.75	0.26	11% (1/9)	4% (1/26)
IL-18	869	519	735	270	0% (0/9)	8% (2/26)
IL-22	9.2	2.9	10.6	2.3	11% (1/9)	23% (6/26)
IL-23	35	36	39	29	0% (0/9)	19% (5/26)
IL-27	0.87	1.31	0.35	0.86	11% (1/9)	4% (1/26)
TNF alpha	24.0	9.1	26.8	6.8	0% (0/9)	12% (3/26)
				*Total mice*	22% (2/9)	35% (9/26)

*Median absolute deviation; ^†^Number of mice with cytokine expression values >3^rd^ quartile + 1.5x interquartile range; in some mice, more than one cytokine was an outlier.

### Comparison of Initiated Colon Tissue From 8-Week-Old Tumor-Free *vs.* Tumor-Bearing *Apc^+/Min-FCCC^* Mice

Given that chemo- and immunopreventive interventions appear to be more effective at reducing tumor incidence and burden in *Apc^+/Min-FCCC^* mice when administered prior to polyp formation *vs.* after development of the first polyp (4, 5), we reasoned that the initiated colonic mucosa of tumor-free *Apc^+/Min-FCCC^* mice might differ from that of *Apc^+/Min-FCCC^* mice bearing at least one macroscopic tumor. Therefore, *Apc^+/Min-FCCC^* mice were classified as ‘tumor-free’ if no lesions were grossly visible within the colon at necropsy, or ‘tumor-bearing’ if at least one lesion was detected. As above, nonneoplastic colon tissue from tumor-free and tumor-bearing *Apc^+/Min-FCCC^* mice were evaluated for perturbations in processes associated with early tumorigenesis, including cellular proliferation (9), stemness (10–12), and EGFR (13, 14) and inflammatory signaling (15, 16).

**Figure 5:**
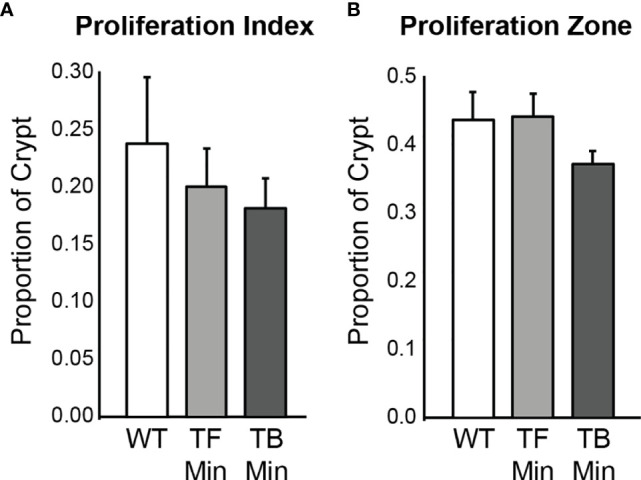
Epithelial cell proliferation in the distal colon of *Apc^+/+-FCCC^* wild type mice (‘WT’; n=4), tumor-free *Apc^+/Min-FCCC^* mice (‘TF Min’; n=6) and tumor-bearing *Apc^+/Min-FCCC^* mice (‘TB Min’; n=6). Bars represent mean ± S.E.M. **(A)** Fold change in proliferative index in tumor-bearing *vs* tumor-free mice = 0.9; Welch’s t-test p = 0.65. **(B)** Fold change in size of the proliferative zone in tumor-bearing *vs* tumor-free mice = 0.8; Welch’s t-test p = 0.15.

The proliferation rates of the initiated epithelium of tumor-free and tumor-bearing *Apc^+/Min-FCCC^* mice were compared. The average proliferative index for initiated, nonneoplastic tissue in tumor-free *Apc^+/Min-FCCC^* mice was 20 ± 3% (Mean ± S.E.M.), whereas the average proliferative index of nonneoplastic tissue from tumor-bearing *Apc^+/Min-FCCC^* mice was 18 ± 3%. The average size of the proliferative zone of nonneoplastic crypts from tumor-free *Apc^+/Min-FCCC^* mice was 44 ± 4% (Mean ± S.E.M.), whereas the average size of the proliferative zone of nonneoplastic crypts from tumor-bearing *Apc^+/Min-FCCC^* mice was 37 ± 2% (Mean ± S.E.M.). Thus, cellular proliferation did not differ in tumor-free *vs* tumor-bearing *Apc^+/Min-FCCC^* mice (Welch’s t-test p = 0.65 for the proliferative index and p = 0.15 for proliferative zone; **Figure 5**).

To examine if pro-tumorigenic gene expression changes in the nonneoplastic colon tissue accompany the formation of a macroscopic tumor, the expression of 8 adenoma-associated genes was compared within the initiated mucosa of tumor-free *vs.* tumor-bearing *Apc^+/Min-FCCC^* mice. To circumvent tumor-elicited effects, only samples located at least 1 cm from the nearest macroscopic colon tumor were analyzed. Overall, the relative mean expression of all genes analyzed was comparable between the nonneoplastic mucosa of tumor-free and tumor-bearing *Apc^+/Min-FCCC^* mice (fold change 0.8 – 1.3, Welch’s t-test p≥0.25; **Table 4** and **Supplemental Table 3**).

**Table 4:** Mean fold change in gene transcription in the initiated colonic mucosa of tumor-bearing *vs* tumor-free *Apc^+/Min-FCCC^* mice after removing outliers.

Gene	Pathway	Fold Change	P-value*
*Ascl2*	Cellular Stemness	1.08	0.75
*Ereg*	EGFR signaling	1.06	0.84
*Grem1*	Cellular stemness	0.77	0.29
*Lgr5*	Cellular stemness	0.79	0.25
*Mmp9*	Inflammation	0.91	0.73
*Muc2*	Cellular differentiation	0.92	0.64
*Ptgs2*	Inflammation	1.25	0.30
*Reg4*	Inflammation	0.95	0.74

*Two-sided Welch’s t-test.

**Figure 6:**
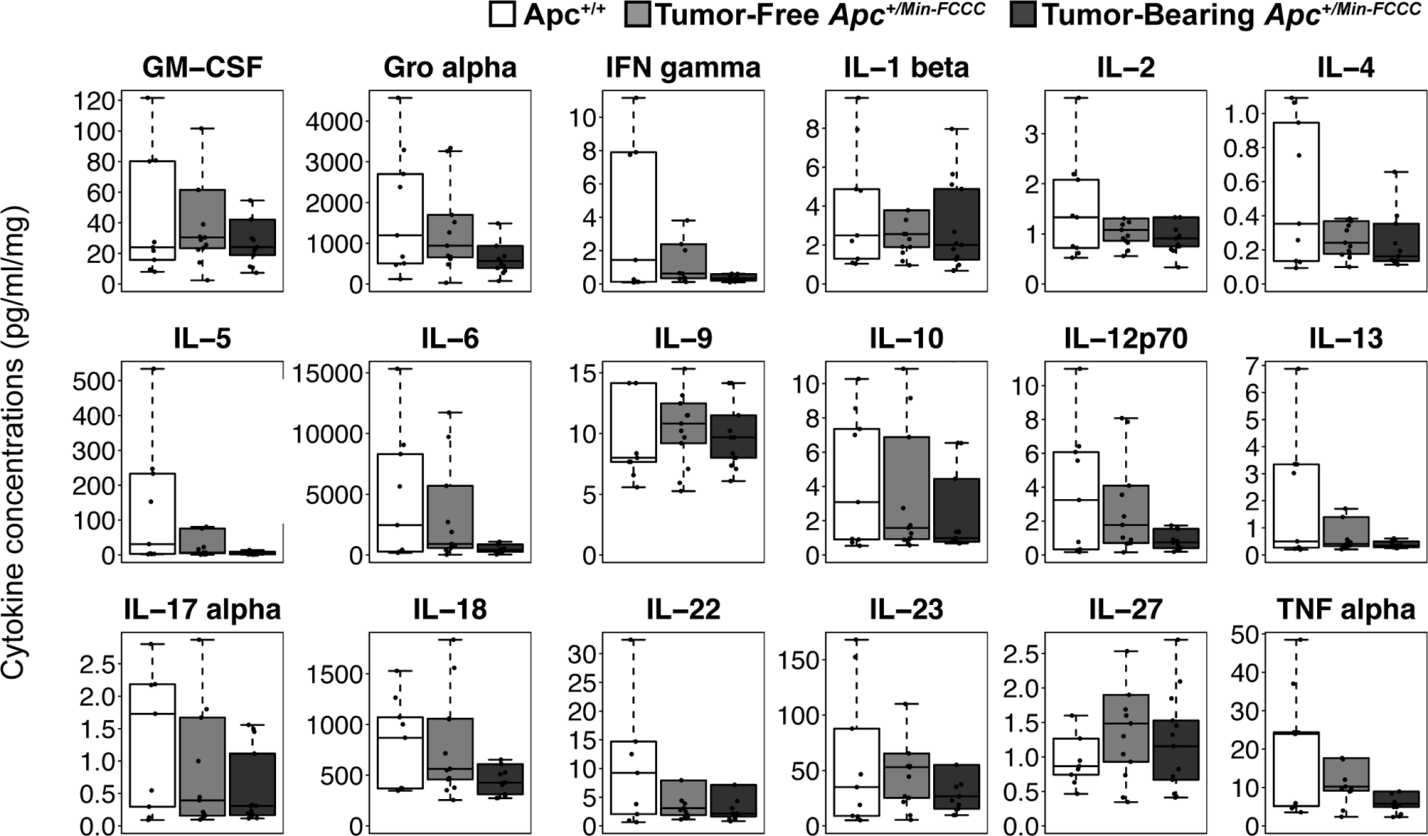
Expression of cytokines involved in Th1, Th2, Th9, and Th17 signaling in nonneoplastic colon tissue from *Apc^+/+-FCCC^* wild type (white; n=9), tumor-free *Apc^+/Min-FCCC^* (light gray; n=13), and tumor-bearing *Apc^+/Min-FCCC^* (dark gray; n=13) mice. Boxplots depict the cytokine concentration in media (pg/ml) normalized to mass of colon tissue (mg) cultured from each mouse. Outlying data points (>3rd quartile + 1.5 x interquartile range) are omitted; all data points are shown in **Supplemental Figure 7**. Fold change in median cytokine expression in tumor-bearing tumor-free = 0.4 – 0.9; Mann Whitney p≥0.06; p=1.00 after adjusting for multiple comparisons.

To assess if increased pro-inflammatory signaling within nonneoplastic colon tissue accompanies the formation of a macroscopic tumor, the secretion of inflammatory cytokines from *ex vivo* segments of nonneoplastic, initiated colon tissue was examined in tumor-free and tumor-bearing *Apc^+/Min-FCCC^* mice. Interestingly, cytokine expression did not differ significantly between the initiated tissues of tumor-bearing *vs* tumor-free *Apc^+/Min-FCCC^* mice (fold change 0.4 – 0.9, Mann-Whitney p≥0.06; **Figure 6** and **Table 5**). However, interindividual variability in the level of cytokine secretion was high, irrespective of the presence or absence of a macroscopic tumor (**Supplemental Figure 7** and **Table 6**); 31% (4/13) of tumor-free *Apc^+/Min-FCCC^* mice and 31% (4/13) of tumor-bearing *Apc^+/Min-FCCC^* mice were defined as outliers due to high expression of one or more cytokines (**Table 6**). Within tumor-bearing animals, the concentration of most cytokines did not correlate with either overall tumor burden (linear model R^2^ ≤ 0.02; **Supplemental Figure 8**) or distance from the nearest detectable tumor (linear model R^2^ ≤ 0.38; **Supplemental Figure 9**). There was a trend toward increasing concentration of cytokine with greater distance from the tumor. This relationship was statistically significant in the case of IL-27 (p=0.03), but significance was lost after correcting for multiple comparisons (adjusted p=0.58).

**Table 5:** Median fold change in secretion of cytokines involved in Th1, Th2, Th9, and Th17 signaling from initiated colon tissues from tumor-bearing *vs* tumor-free *Apc^+/Min-FCCC^* mice.

Cytokine	Fold change	P-value*
GM-CSF	0.79	0.34
Gro alpha	0.60	0.11
IFN gamma	0.53	0.11
IL-1 beta	0.78	0.42
IL-2	0.84	0.47
IL-4	0.67	0.23
IL-5	0.44	0.29
IL-6	0.45	0.10
IL-9	0.89	0.57
IL-10	0.62	0.43
IL-12p70	0.42	0.13
IL-13	0.84	0.38
IL-17a	0.78	0.66
IL-18	0.76	0.22
IL-22	0.70	0.57
IL-23	0.50	0.56
IL-27	0.78	0.37
TNF alpha	0.56	0.06

*Uncorrected Mann-Whitney test; p=1.00 after correcting for multiple comparisons.

**Table 6:** Intra-group variability in secretion of cytokines involved in Th1, Th2, Th9, and Th17 signaling from initiated colon tissue from tumor-free and tumor-bearing *Apc^+/Min-FCCC^* mice.

Cytokine	Median Concentration (pg/ml/mg)		MAD* Expression		Proportion of mice with outlying expression^†^	
	Tumor Free	Tumor Bearing	Tumor Free	Tumor Bearing	Tumor Free	Tumor Bearing
GM-CSF	30.4	24.2	12.7	19.0	15% (2/13)	15% (2/13)
Gro alpha	941	566	683	358	15% (2/13)	15% (2/13)
IFN gamma	0.63	0.33	0.64	0.23	15% (2/13)	15% (2/13)
IL-1 beta	2.56	2.00	1.41	1.52	23% (3/13)	0% (0/13)
IL-2	1.58	0.98	1.34	0.45	8% (1/13)	8% (1/13)
IL-4	1.77	0.74	1.64	0.69	8% (1/13)	15% (2/13)
IL-5	0.41	0.34	0.23	0.10	15% (2/13)	15% (2/13)
IL-6	0.39	0.31	0.41	0.22	8% (1/13)	0% (0/13)
IL-9	562	428	313	201	8% (1/13)	15% (2/13)
IL-10	1.08	0.91	0.34	0.32	23% (3/13)	15% (2/13)
IL-12p70	3.08	2.14	2.30	1.51	23% (3/13)	23% (3/13)
IL-13	53.0	26.6	41.0	18.0	15% (2/13)	23% (3/13)
IL-17a	1.49	1.16	0.82	0.72	15% (2/13)	0% (0/13)
IL-18	0.24	0.16	0.13	0.07	15% (2/13)	8% (1/13)
IL-22	6.74	2.96	8.91	3.09	15% (2/13)	15% (2/13)
IL-23	905	410	1313	374	8% (1/13)	15% (2/13)
IL-27	10.8	9.68	2.46	2.69	8% (1/13)	8% (1/13)
TNF alpha	10.2	5.73	9.30	4.06	23% (3/13)	23% (3/13)
				*Total mice*	31% (4/13)	31% (4/13)

*Median absolute deviation; ^†^Number of mice with cytokine expression values >3^rd^ quartile + 1.5x interquartile range; in some mice, more than one cytokine was an outlier.

## Discussion

This study provides the first highly controlled characterization of early carcinogenic processes within the nonneoplastic colon of *Apc^Min^* mice: (1) prior to tissue initiation, (2) after initiation but prior to adenoma formation, and (3) after initiation and adenoma formation (**Figure 1**). To maximize the benefits of preventive intervention while minimizing the risk of side effects, it is critical to identify the ideal time to intervene. The *Apc^+/Min-FCCC^* mouse model is a powerful system with which to study the timing of molecular events during early colon tumorigenesis. A methodical approach was employed to characterize transformational processes in the colonic mucosa. Confounding variables from genetics and cage environment were controlled through block randomization of age-matched litters of *Apc^+/Min-FCCC^* mice and wild type animals. Small (2 mm) regions of only the distal colon were examined to control for variation in cellular proliferation, gene expression, and cytokine production along the length of the colon. The resulting data establish the baseline levels of cellular proliferation, relative gene expression, and cytokine production in the nonneoplastic mucosa of young adult mice (regardless of *Apc* mutational status) and may inform the design of new preclinical prevention studies.

To determine the impact of a heterozygous *APC* mutation on molecular characteristics associated with early tumorigenesis, “initiated”, at-risk nonneoplastic tissue from the distal colon of *Apc^+/Min-FCCC^* mice was compared to uninitiated tissue from the distal colon of wild type littermates. Interestingly, no significant differences were identified between nonneoplastic tissues from tumor-bearing *Apc^+/Min-FCCC^*, tumor-free *Apc^+/Min-FCCC^*, and wild type animals, with minimal changes observed in cellular proliferation, transcription of select genes related to cellular stemness, EGFR signaling, and inflammation, or secretion of select cytokines indicative of an inflamed microenvironment. Thus, we did not find evidence that the 8 genes or 18 cytokines examined in the present study would be sufficient targets for preventive intervention initiated prior to the formation of the first colon tumor in individuals who are at elevated risk of developing CRC.

Other groups have observed that a single truncating mutation in *Apc* (as seen in *Apc^+/Min-FCCC^* mice) may increase Wnt signaling at the base of the nonneoplastic colon crypt; enhanced signaling may then drive an expansion of colon epithelial stem cells, increase cellular proliferation along the length of the crypt (27), and ultimately promote adenoma formation (30, 56). In the present study, however, no changes in gene expression or proliferation rates were identified in the nonneoplastic epithelium among wild type, tumor-free *Apc^+/Min-FCCC^*, and tumor-bearing *Apc^+/Min-FCCC^* mice. It is possible that the initiating mutation in *Apc^+/Min-FCCC^* mice was insufficient to drive even modest changes in gene transcription within the colons of 8-week-old *Apc^+/Min-FCCC^* mice. Notably, however, only a small number of genes were examined in this study. Thus, a more comprehensive RNA-Seq analysis may reveal transcriptional changes in other genes. Alternatively, it is possible that gene expression and proliferative changes arise within a specific cellular compartment of the crypt. Since the entire mucosa was examined in the present study, molecular alterations specific to a subpopulation of cells in each crypt would likely be masked. Interestingly, the transcript levels of genes associated with Wnt signaling and cellular stemness, including *Ascl2* and *Lgr5*, were slightly elevated in the nonneoplastic mucosa of *Apc^+/Min-FCCC^ vs*. wild type mice (**Table 1**). Although the differences were modest and did not reach statistical significance, these trends may indicate subtle changes within the stem cell compartment of young adult *Apc^+/Min-FCCC^* mice compared to wild type animals. This shift is consistent with the observation that chemo- and immunoprevention strategies that target the stem cell compartment, including atorvastatin and a vaccine against *Ascl2*, are most effective when administered prior to tumor formation (4, 5).

Inflammation drives tumorigenesis within the colon, and may be an early event in the initiation of many sporadic and hereditary colon cancers (57–60). Mutations in *Apc* are associated with shifts in the gut microbiome, which may in turn induce inflammation (61). In the present study, no changes in cytokine secretion or the expression of inflammation-associated genes were identified in the nonneoplastic epithelium among 8-week-old wild type, tumor-free *Apc^+/Min-FCCC^*, and tumor-bearing *Apc^+/Min-FCCC^* mice. However, pro-inflammatory signaling may become more pronounced at a timepoint later than what was examined in this study. Consistent with this hypothesis, the production of inflammatory cytokines increases significantly within the initiated intestinal epithelium of C57BL6 *Apc^Min^* mice as animals approach the end of their life span, as compared to normal intestinal tissue from age-matched wild type C57BL6 mice (albeit animals from a different colony), and correlates with the number of large adenomas in each animal (25). Notably, expression of MCP-1, IL-1β, IL-6 and TNF-alpha within the small intestine of *Apc^Min^* mice did not differ from that of age-matched wild type mice at 8 weeks of age, but did increase significantly by 12-16 weeks of age (25).

In the present study, some animals (irrespective of *Apc* mutational status) had higher baseline production of inflammatory cytokines within the colonic mucosa than others at 8 weeks of age. The biological variability among mice within each group was typically larger than the average differences between groups. This was unexpected as extensive measures were taken to standardize experimental conditions across the groups. The source of this variability remains unclear; no single genetic or environmental factor, including litter, age, or cage environment, appeared to account for the large inter-individual variability. Regardless of what causes this interindividual heterogeneity, it must occur early and before the animals reach sexual maturity at 8 weeks of age. This heterogeneity may contribute to the phenotypic differences observed at older ages. Notably, in a separate study from this group, *Apc^+/Min-FCCC^* mice that did not have colon tumors at 6-8 weeks of age developed significantly fewer tumors by 20-22 weeks of age (1.8 ± 1.3 Mean ± S.D.) than animals that had a tumor at 6-8 weeks of age (6.4 ± 3.9; Mean ± S.D.; p=0.002) (4). These data suggest that the number of colon tumors that *Apc^+/Min-FCCC^* mice develop, while highly variable, is influenced by events that occur early in the lifespan of the animal (by or before 8 weeks of age).

Given that inflammation promotes tumor formation, growth, and progression following loss of *Apc* (51, 57, 62, 63), longitudinal studies are needed in the future to determine if *Apc^+/Min-FCCC^* mice with high baseline levels of cytokine activity represent a subpopulation that is at highest risk of developing colorectal tumors. Colon tissue biopsies could be collected at 8 weeks of age to assess cytokine production within nonneoplastic colon tissue prior to tumor formation. However, tissue damage from a biopsy might trigger an inflammatory response, thus confounding the analyses. Alternatively, cytokine levels could be measured in proxy samples, such as serum or feces (64, 65). Ultimately, high baseline inflammatory signaling in the normal colonic mucosa, serum, or feces of healthy young adults may prove to be a novel biomarker of colon cancer risk.

In conclusion, the data from the present study provide valuable insight into the basal levels of cellular proliferation and cytokine production in the nonneoplastic mucosa of young adult mice. These data can be used to inform the design of future preclinical prevention studies. Notably, a high degree of variability was observed in the baseline levels of cytokine production in age-matched, genetically identical animals. These subpopulations of animals with elevated cytokine signaling in the nonneoplastic colonic mucosa may represent a high-risk group that would benefit from earlier intervention. Further study is needed to better understand the contribution of high baseline cytokine production within the nonneoplastic colonic mucosa to long-term colon cancer risk.

## Data Availability Statement

The raw data supporting the conclusions of this article will be made available upon request by the authors, without undue reservation.

## Ethics Statement

The animal study was reviewed and approved by The Institutional Animal Care and Use Committee at the Fox Chase Cancer Center.

## Author Contributions

AL and MC contributed to the conception and design of the study. KH performed animal husbandry. AL, KH, EK, and HH conducted the colonoscopies. AL, MC, KH, and LV performed necropsies. AL performed the remaining experiments. AL and KD performed the statistical analyses. AL wrote the first draft of the manuscript, and MC wrote sections of the manuscript. All authors contributed to the article and approved the submitted version.

## Funding

This research was supported by grants from the National Cancer Institute, National Institutes of Health (P30 CA006927 and T32 CA009035), and by the Timothy and Aurora Hughes Colon Cancer Research Fund.

## Conflict of Interest

The authors declare that the research was conducted in the absence of any commercial or financial relationships that could be construed as a potential conflict of interest.

## Publisher’s Note

All claims expressed in this article are solely those of the authors and do not necessarily represent those of their affiliated organizations, or those of the publisher, the editors and the reviewers. Any product that may be evaluated in this article, or claim that may be made by its manufacturer, is not guaranteed or endorsed by the publisher.
