# Supplementary material for: High Variability in Cellular Proliferation, Gene Expression, and Cytokine Production in the Nonneoplastic Colonic Epithelium of Young Apc+/Min-FCCC Mice
Source: Front Oncol. 2021 Aug 27;11:705562. doi: 10.3389/fonc.2021.705562 (PMC8429936; doi:10.3389/fonc.2021.705562)
Supplement: Supplementary file 1 [file DataSheet_1.docx]

Supplementary Material

# Supplementary Figures


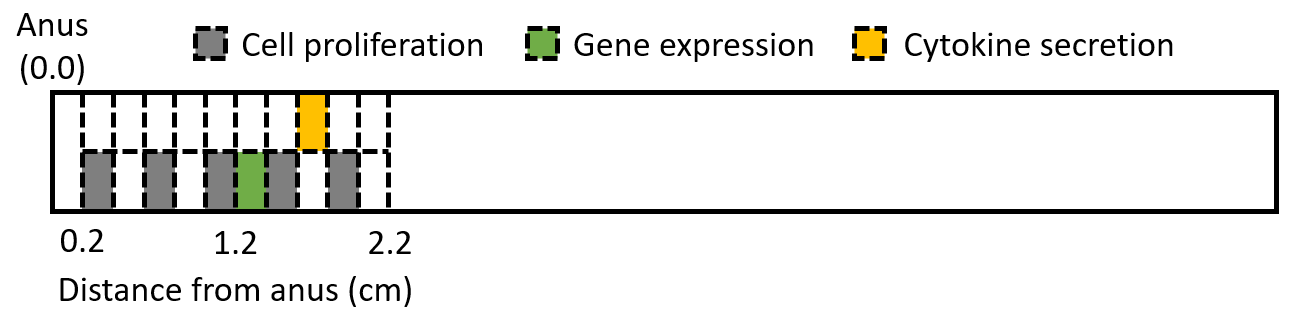


**Supplementary Figure 1.** The distal 2.2 cm of colon were cut in half lengthwise and each half was divided into 2 mm sections (dotted lines). Alternating pieces from one side were snap frozen in liquid nitrogen or placed in media for *ex vivo* cytokine analysis. Alternating pieces from the other side were frozen in OCT for downstream gene expression analyses or fixed in 4% paraformaldehyde prior to freezing in OCT for cellular proliferation analyses. Data described in this paper were obtained from the following regions of the colon: proliferation data were derived from five 2 mm sections (.2, 0.6, 1.0, 1.4, and 1.8 mm from the anus; gray boxes); gene expression data were derived from a single 2 mm section (1.2 cm from the anus; green box); and cytokine secretion data were derived from a single 2 mm section (1.6 cm from the anus; yellow box). Any tissue section that contained a macroscopic colon tumor was excluded from analyses.


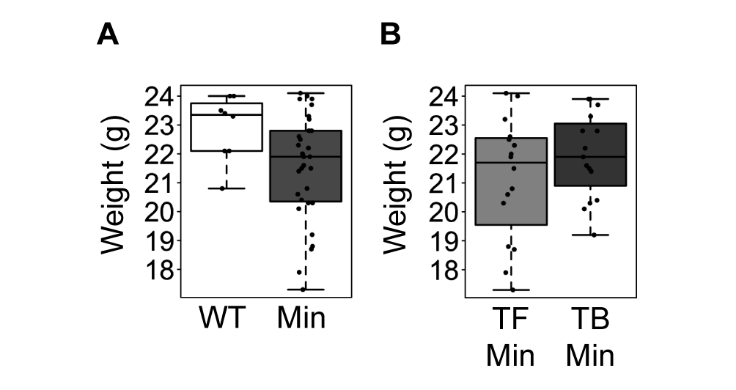


**Supplementary Figure 2.** Supplemental Figure 2. Mouse weights at 8 weeks of age by genotype and tumor status. **(A)** *Apc^+/Min-FCCC^* mice (Min) weigh slightly less than wild type (WT) at 8 weeks of age; median fold change = 0.94; Mann-Whitney test p=0.04. **(B)** *Apc^+/Min-FCCC^* mice with macroscopic tumors in the colon at 8 weeks of age (TB Min) are similar in size to tumor-free *Apc^+/Min-FCCC^* mice (TF Min); median fold change = 1.01; Mann-Whitney test p=0.43.


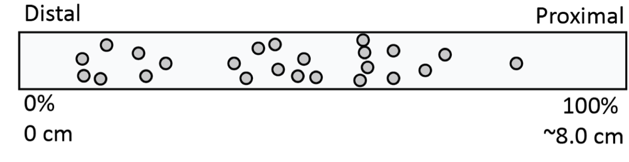


**Supplementary Figure 3**. Tumor location in the colons of tumor-bearing *Apc^+/Min-FCCC^* mice at 8 weeks of age. The location of 24 tumors from 15 tumor-bearing *Apc^+/Min-FCCC^* mice are plotted based on location in the colon relative to the anus. Approximately 1/3 of tumors are located in the distal third of the colon, and most of the remaining 2/3 of tumors are located in the medial third of the colon.


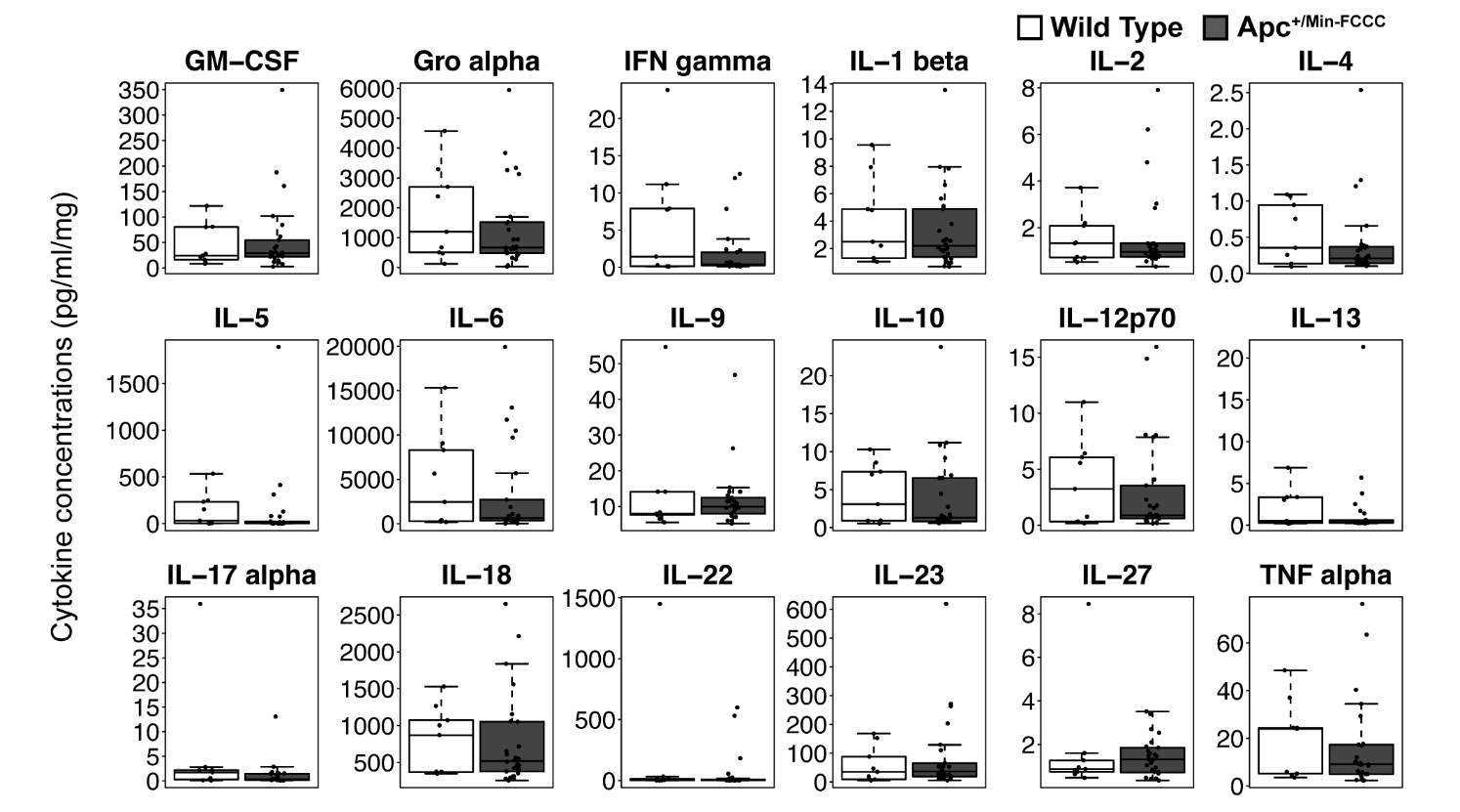


**Supplementary Figure 4**. Cytokines involved in Th1, Th2, Th9, and Th17 signaling are expressed at similar levels from nonneoplastic colon tissues from *Apc^+/Min-FCCC^* and wild type mice. Boxplots of cytokine concentration in media (pg/ml) normalized to mass of colon tissue (mg) from wild type mice (white; n=9) and *Apc^+/Min-FCCC^* mice (gray; n=26). Median fold change = 0.2 – 1.5; Mann Whitney p≥0.17; p=1.00 after adjusting for multiple comparisons.


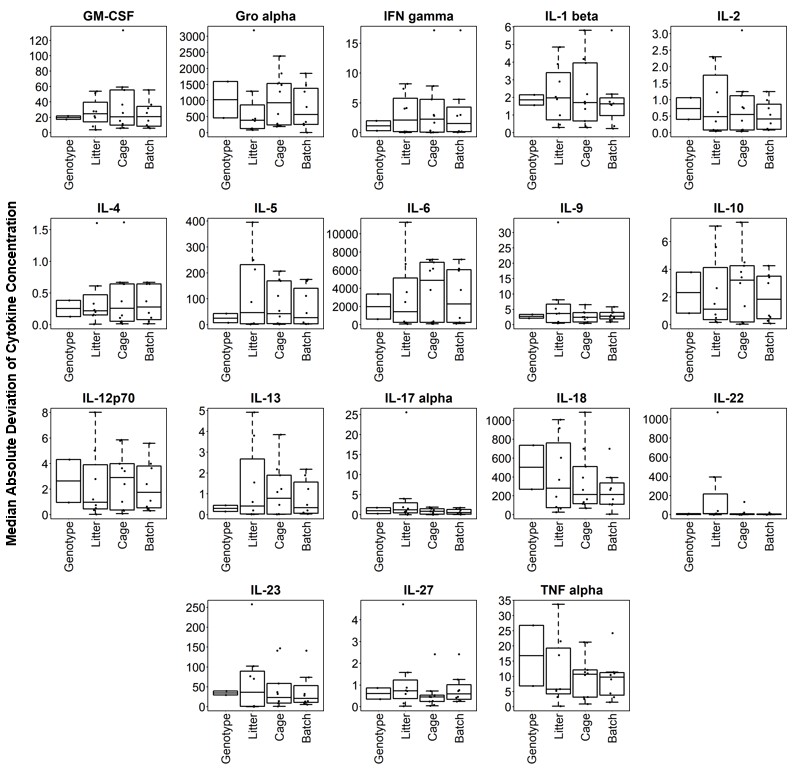


**Supplementary Figure 5.** Median absolute deviation (MAD) of cytokine expression was high regardless of whether animals were from the same genotype, litter, cage, or batch. The variation in cytokine expression was estimated by calculating the MAD score for each genotype (n=2), litter (n=8), cage (n=10), and batch (n=8) that contained more than a single animal. Boxplots demonstrate the wide range in MAD scores found in each category; many individual litters, cages, and batches exhibited more variability than was observed within each genotype.


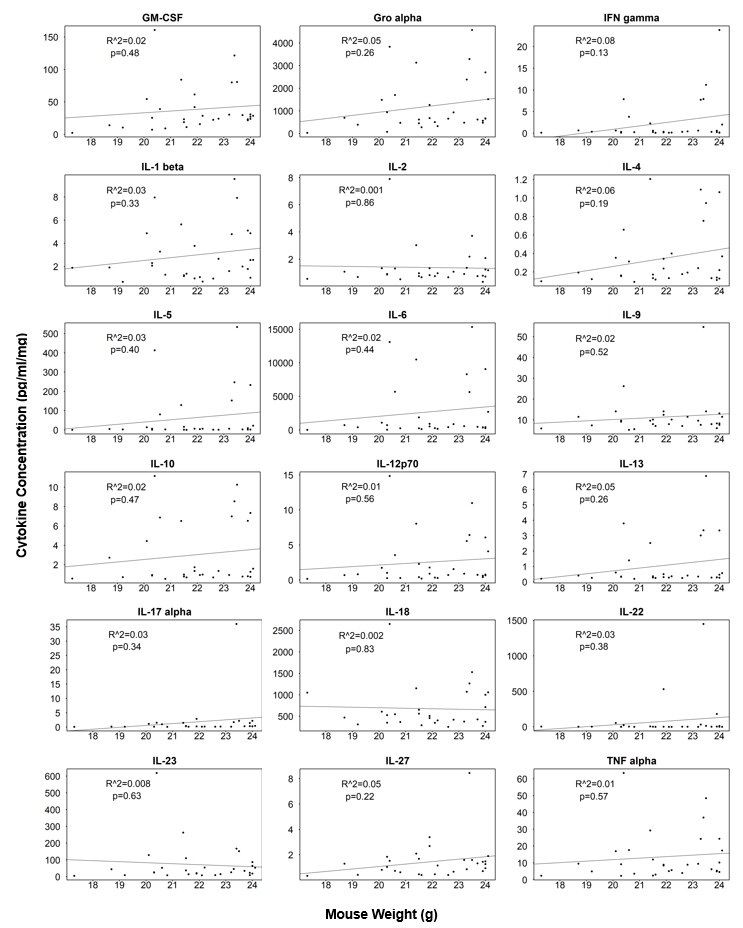


**Supplementary Figure 6.** Cytokines are secreted at similar levels from nonneoplastic colon tissues from wild type (n=9) and *Apc^+/Min-FCCC^* mice (n=26) regardless of the mass of the animal. Scatterplots of cytokine concentration in media (pg/ml) normalized to mass of colon tissue (mg) plotted by mass of the animal (g). Best fit linear line is plotted with the data; R2≤0.08; p≥0.13; p=1.00 after adjusting for multiple comparisons.


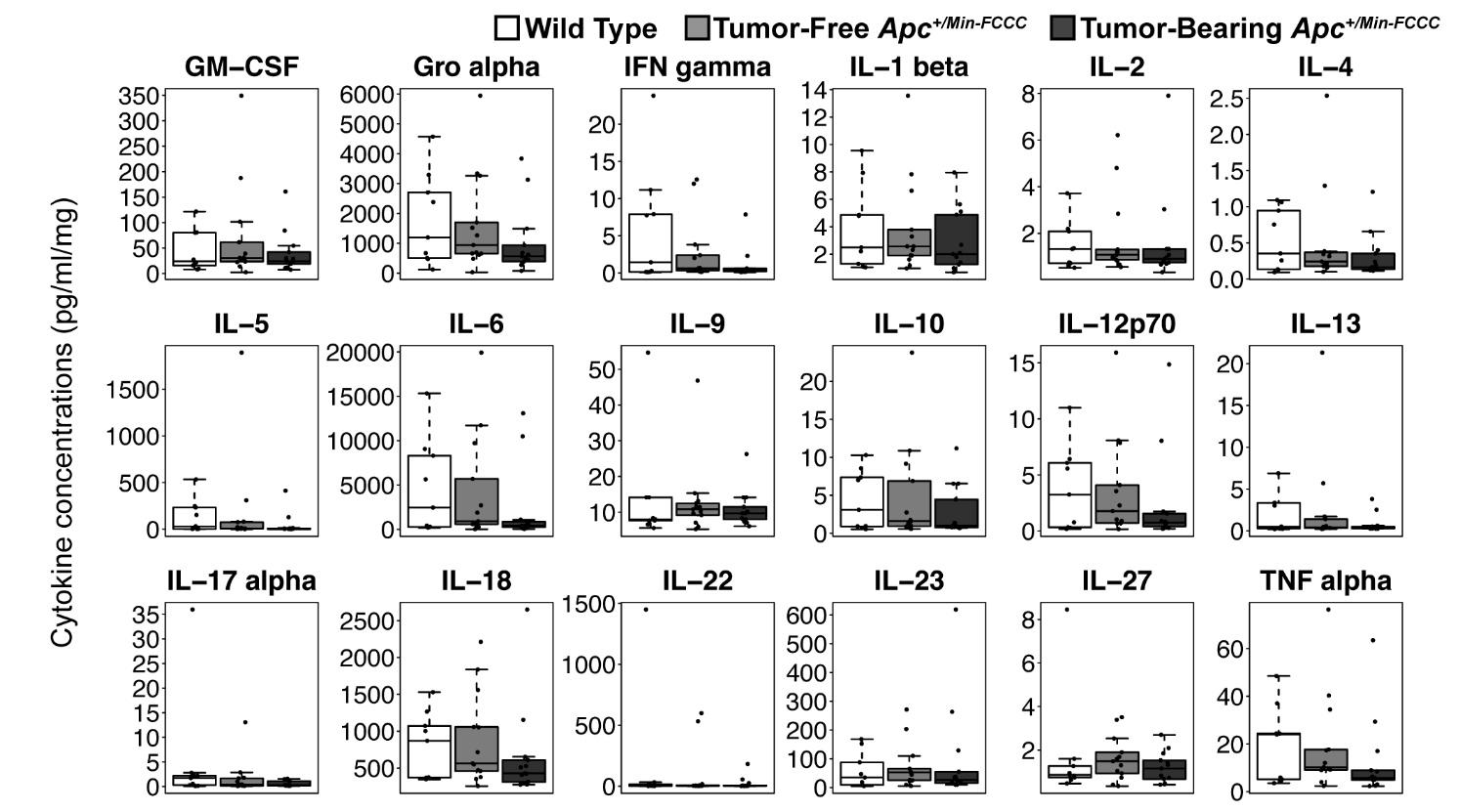


**Supplementary Figure 7.** Cytokines involved in Th1, Th2, Th9, and Th17 signaling are expressed at similar levels from nonneoplastic colon tissues of wild type (white; n=9), tumor-free *Apc^+/Min-FCCC^* (light gray; n=13), and tumor-bearing *Apc^+/Min-FCCC^* (dark gray; n=13) mice. Boxplots of cytokine concentration in media (pg/ml) normalized to mass of colon tissue (mg) cultured from each mouse. Median fold change of tumor-bearing vs tumor-free = 0.4 – 0.9; Mann Whitney p≥0.06; p=1.00 after adjusting for multiple comparisons.


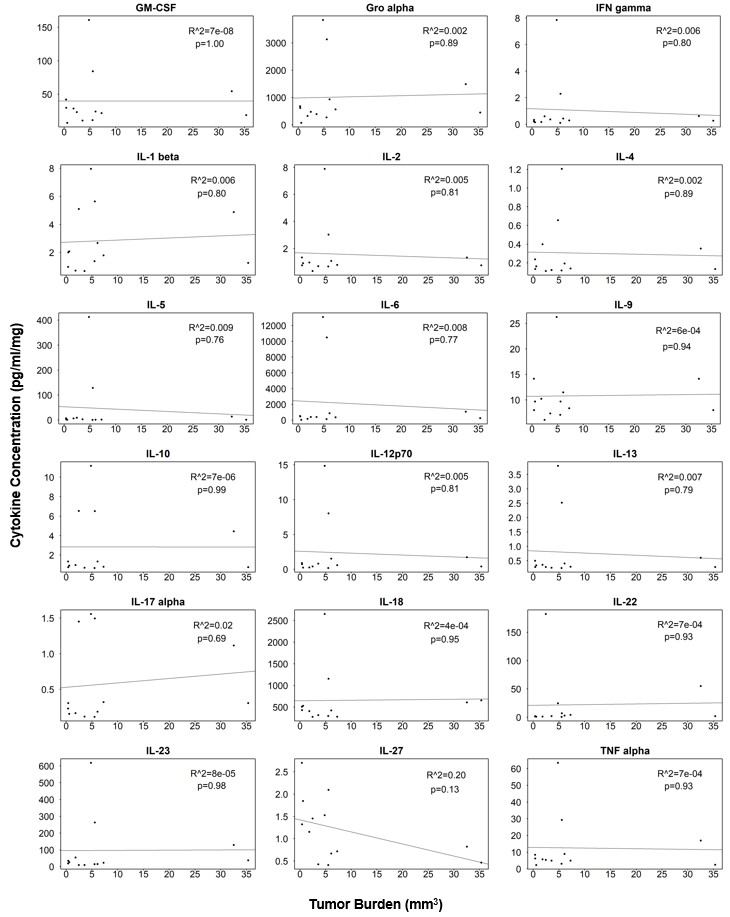


**Supplementary Figure 8.** Cytokines are secreted at similar levels from initiated colon tissues from *Apc^+/Min-FCCC^* mice with colon tumors regardless of the total colon tumor burden. Scatterplots of cytokine concentration in media (pg/ml) normalized to mass of colon tissue (mg) from *Apc^+/Min-FCCC^* mice with colon tumors (n=13) plotted by total estimated volume (mm^3^) of all macroscopic tumors in the colon. Best fit linear line is plotted with the data; R2≤0.02; p≥0.13; p=1.00 after adjusting for multiple comparisons.


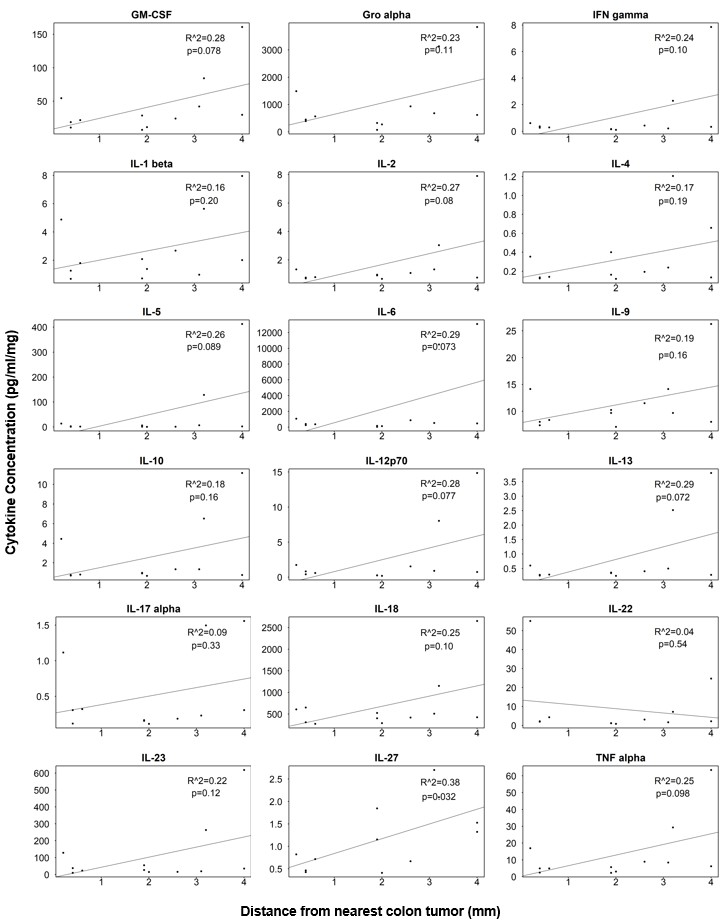


**Supplementary Figure 9.** Cytokines are secreted at similar levels from initiated colon tissues from *Apc^+/Min-FCCC^* mice with colon tumors regardless of the total colon tumor burden. Scatterplots of cytokine concentration in media (pg/ml) normalized to mass of colon tissue (mg) from *Apc^+/Min-FCCC^* mice with colon tumors (n=13) plotted by distance (mm) from the nearest macroscopic colon tumor. Best fit linear line is plotted with the data; R^2^≤0.38; p≥0.03 (p≥0.58 after adjusting for multiple comparisons).

# Supplementary Tables

**Supplementary Table 1.** Primer sequences used for quantitative PCR.

| **Gene** | **Direction** | **Primer Sequence** |
| --- | --- | --- |
| *Ascl2* | Forward | GAAGGTGCAAACGTCCACTT |
|  | Reverse | TCCATCAAGCTTGCATTCAG |
| *Ereg* | Forward | AAGCTGCACCGAGAAAGAAG |
|  | Reverse | GGATCACGGTTGTGCTGATA |
| *Grem1* | Forward | AGCCTAAGATGAGCGCC |
|  | Reverse | TTACTAGGCAGATGGCCACA |
| *Lgr5* | Forward | CGGAGGAAGCGCTACAGAAT |
|  | Reverse | CTGGGTGGCACGTAGCTGAT |
| *Mmp9* | Forward | AGACGACATAGACGGCATCC |
|  | Reverse | CTGTCGGCTGTGGTTCAGT |
| *Muc2* | Forward | CAAGGGCTCGGAACTCCAG |
|  | Reverse | CCAGGGAATCGGTAGACATCG |
| *Ptgs2* | Forward | TGCACTATGGTTACAAAAGCTGG |
|  | Reverse | TCAGGAAGCTCCTTATTTCCCTT |
| *Reg4* | Forward | AAAACTGGAACGGGCTCTGAGG |
|  | Reverse | GCTGGTAGATGCTTGATTCTTGCTC |

**Supplementary Table 2.** Mean fold change in gene transcription in the nonneoplastic mucosa of  *Apc^+/Min-FCCC^* vs wild type mice.

| **Gene** | **Pathway** | **Fold Change** | **P-value*** |
| --- | --- | --- | --- |
| *Ascl2* | Cellular Stemness | 0.92 | 0.82 |
| *Ereg* | EGFR signaling | 1.11 | 0.76 |
| *Grem1* | Cellular stemness | 1.02 | 0.96 |
| *Lgr5* | Cellular stemness | 0.98 | 0.96 |
| *Mmp9* | Inflammation | 0.97 | 0.90 |
| *Muc2* | Cellular differentiation | 1.37 | 0.23 |
| *Ptgs2* | Inflammation | 0.90 | 0.65 |
| *Reg4* | Inflammation | 0.79 | 0.34 |
| *Two-sided Welch’s t-Test | | | |

**Supplementary Table 3.** Mean fold change in gene transcription in the nonneoplastic mucosa of tumor-bearing vs tumor-free *Apc^+/Min-FCCC^* mice.

| **Gene** | **Pathway** | **Fold Change** | **P-value*** |
| --- | --- | --- | --- |
| *Ascl2* | Cellular Stemness | 1.76 | 0.33 |
| *Ereg* | EGFR signaling | 1.52 | 0.38 |
| *Grem1* | Cellular stemness | 1.30 | 0.66 |
| *Lgr5* | Cellular stemness | 1.60 | 0.54 |
| *Mmp9* | Inflammation | 1.18 | 0.66 |
| *Muc2* | Cellular differentiation | 0.92 | 0.64 |
| *Ptgs2* | Inflammation | 1.06 | 0.81 |
| *Reg4* | Inflammation | 1.25 | 0.50 |
| *Two-sided Welch’s t-Test | | | |
